# Supplementary material for: Bronchoscopic Endobronchial Valve Therapy for Persistent Air Leaks in COVID-19 Patients Requiring Veno-Venous Extracorporeal Membrane Oxygenation
Source: J Clin Med. 2023 Feb 8;12(4):1348. doi: 10.3390/jcm12041348 (PMC9962378; doi:10.3390/jcm12041348)
Supplement: Supplementary file 1 [file jcm-12-01348-s001.zip › jcm-2164595-supplementary.pdf]

Bronchoscopic Endobronchial Valve Therapy for Persistent Air Leaks in COVID-19 Patients Requiring Veno-Venous Extracorporeal Membrane Oxygenation

Barbara Ficial, Stephen Whebell, Daniel Taylor, Rita Fernández-Garda, Lawrence Okiror,

and Christopher I. S. Meadows

Supplementary Material

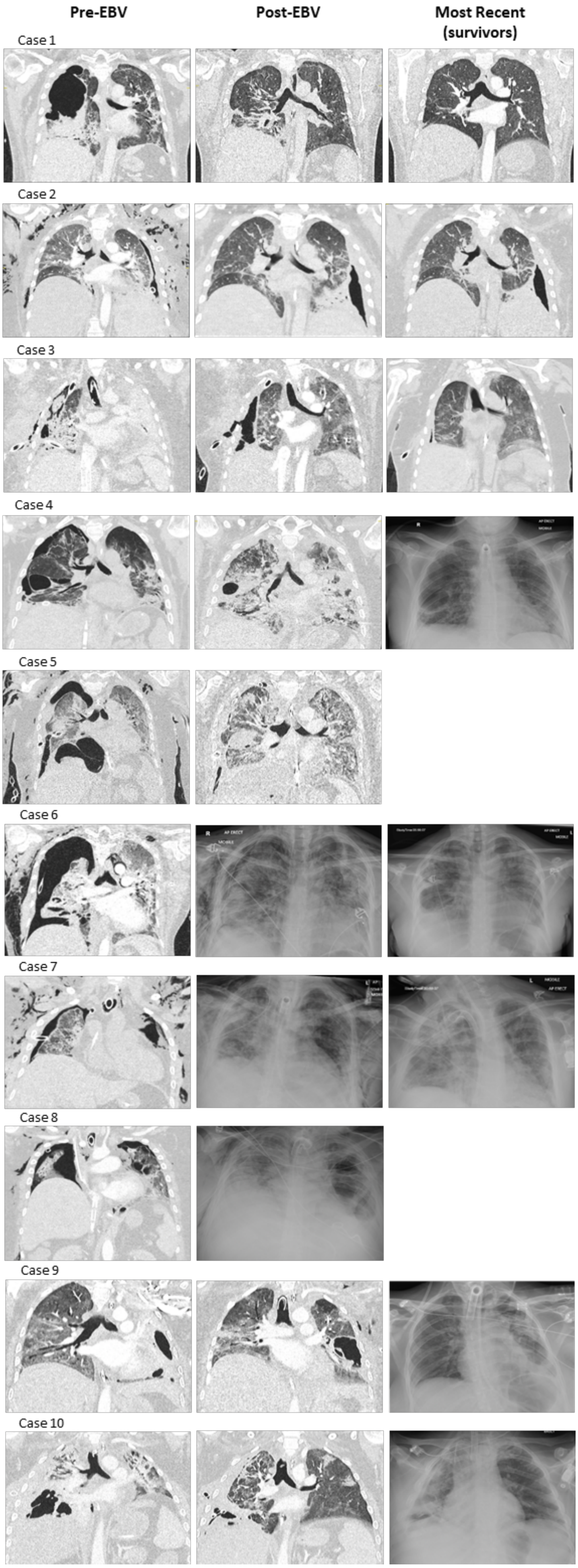

Supplementary Figure SA: Imaging of all cases before EBV placement, after EBV placement and the most recent imaging available for survivors
CT imaging used where available, plain film x-ray presented otherwise
EBV – endobronchial valve
